# Immune microenvironment biomarkers linked to ferroptosis in clear cell renal cell carcinoma

**DOI:** 10.3389/fgene.2026.1938003

**Published:** 2026-10-28

**Authors:** Peihua Li, Xin Liu, Guanchong Li

**Affiliations:** 1 Department of Urology, Chengyang District People’s Hospital, Qingdao, Shandong, China; 2 Department of Oncology, Huai’an Clinical Medical College of Jiangsu University (Huai’an Hospital of Huai’an City), Huai’an, Jiangsu, China; 3 Department of Human Anatomy, Qilu Medical University, Zibo, Shandong, China

**Keywords:** biomarkers, clear cell renal cell carcinoma, ferroptosis, immune infiltration, immune microenvironment, single-cell RNA sequencing

## Abstract

**Background:**

Clear cell renal cell carcinoma (ccRCC) is characterized by marked metabolic reprogramming and a highly heterogeneous immune microenvironment. Ferroptosis is an iron-dependent form of regulated cell death driven by lipid peroxidation and has been implicated in ccRCC biology. This study aimed to identify immune-microenvironment-associated hub genes in ccRCC, examine their relationship with ferroptosis-related transcriptional changes, and evaluate their diagnostic and cellular-expression characteristics.

**Methods:**

Public multi-omics data were analyzed using GSE53757 (72 ccRCC and 72 matched normal kidney tissues), GSE159115 single-cell RNA-sequencing data, and TCGA-KIRC (542 primary tumors and 72 normal kidney tissues). Differential expression, hierarchical co-expression clustering, ferroptosis-gene enrichment, protein-protein interaction analysis, MCP-counter immune scoring, single-cell localization, ROC analysis, and exploratory survival analysis were performed. In addition, *in vitro* qRT-PCR experiments were performed for SLC7A11, GPX4, ACSL4, and HMOX1 in RPTEC and 786-O cells and for CCR5, CSF1R, and CD86 in macrophage cultures, with three biological replicates per group, using the complete ΔCt/ΔΔCt/2^−ΔΔCt^ validation workflow.

**Results:**

GSE53757 identified 8,650 differentially expressed genes, including significant enrichment of detected FerrDb ferroptosis genes (hypergeometric p = 2.3 × 10^−3^). CCR5, CSF1R, CD86, CD8A, and LCP2 were significantly upregulated and clustered within the ccRCC-associated M03 immune co-expression module. MCP-counter and single-cell analyses localized these signals predominantly to macrophage and T/NK-cell compartments. TCGA-KIRC independently confirmed significant upregulation and diagnostic discrimination (AUC 0.862–0.948; five-gene panel AUC = 0.932), whereas the panel showed no independent overall-survival association. *In vitro* qRT-PCR measurements confirmed higher relative expression of SLC7A11, GPX4, ACSL4, and HMOX1 in 786-O than in RPTEC cells, with fold changes of 2.55, 1.58, 1.70, and 2.30, respectively; CCR5, CSF1R, and CD86 were also higher in macrophages exposed to 786-O-conditioned medium than in M0 macrophages.

**Conclusion:**

CCR5, CSF1R, CD86, CD8A, and LCP2 are best interpreted as immune-microenvironment-associated hub genes in ccRCC rather than canonical ferroptosis drivers. Their association with ferroptosis is supported at the pathway and co-expression levels, while direct regulation of iron metabolism, lipid peroxidation, or the GPX4/SLC7A11 axis is not established by the present analyses. The multi-dataset evidence supports their potential diagnostic and immune-stratification value in ccRCC.

## 1 Introduction

Clear cell renal cell carcinoma (ccRCC) is the predominant histological subtype of renal cell carcinoma and is characterized by extensive metabolic reprogramming, constitutive angiogenic signaling, and a highly heterogeneous tumor immune microenvironment. The clinical management of advanced ccRCC has been transformed by immune checkpoint blockade and anti-angiogenic combinations, but primary and acquired resistance remain common and underscore the need for biologically grounded biomarkers that can capture both tumor-cell state and immune contexture (Motzer et al., 2018).

Ferroptosis is a non-apoptotic, iron-dependent form of regulated cell death driven by phospholipid peroxidation and failure of cellular antioxidant defenses (Dixon et al., 2012). The canonical ferroptosis network includes iron handling, polyunsaturated-fatty-acid metabolism, glutathione synthesis, and the GPX4/SLC7A11 antioxidant axis. ccRCC is particularly relevant to ferroptosis research because its hallmark VHL-HIF metabolic phenotype alters lipid storage, redox balance, mitochondrial metabolism, and oxygen sensing, creating a cellular state in which lipid-peroxide homeostasis may have both biological and therapeutic importance (Jiang et al., 2021).

The immune microenvironment is equally central to ccRCC biology. Clinical trials of nivolumab plus ipilimumab and pembrolizumab plus axitinib established immune-based treatment as a major therapeutic strategy in advanced renal cell carcinoma (Rini et al., 2019). Nevertheless, clinical response cannot be inferred from the presence of immune cells alone. Genomic and transcriptomic studies indicate that immune infiltration, somatic alterations, angiogenic programs, and inflammatory signaling jointly shape sensitivity or resistance to PD-1-directed therapy (Braun et al., 2020). This complexity motivates biomarker strategies that integrate tumor metabolism with cell-type-resolved immune information rather than relying on a single bulk-expression signal.

Transcriptomic profiling has demonstrated that ccRCC tumors can be stratified into biologically distinct microenvironmental states, including immune-infiltrated and angiogenesis-dominant patterns (Hakimi et al., 2019). Several of the genes identified in the present study are established immune-lineage genes rather than canonical ferroptosis enzymes. CCR5, for example, has been implicated in the immunosuppressive microenvironment of BAP1-altered ccRCC, where CCR5 blockade can reshape antitumor immunity (Zhou et al., 2020). Similarly, the CCL5-CCR5 axis links tumor-associated macrophages, T-cell dysfunction, and ccRCC progression, highlighting how chemokine signaling can connect immune-cell recruitment with tumor behavior (Xu et al., 2022).

CD8-positive T-cell infiltration also shows marked spatial and functional heterogeneity in ccRCC. Recent tissue-based work has shown that the density, exhaustion state, and spatial proximity of CD8-positive T cells can carry different biological implications, emphasizing that bulk CD8A abundance should be interpreted primarily as a marker of immune-cell composition rather than as a tumor-cell-intrinsic pathway readout (Shapiro et al., 2024). At the same time, single-cell studies of ccRCC have identified ferroptosis-associated transcriptional heterogeneity across tumor and immune compartments, providing a rationale for examining whether ferroptosis-related remodeling and immune-cell programs co-occur within the same disease ecosystem (Zhang et al., 2023).

Accordingly, the present study distinguishes canonical ferroptosis regulators from immune-microenvironment-associated hub genes that coexist with ferroptosis-related transcriptional remodeling. We integrated GSE53757 bulk transcriptomics, hierarchical co-expression clustering, functional enrichment, PPI analysis, MCP-counter immune scoring, GSE159115 single-cell RNA sequencing, and TCGA-KIRC external validation to characterize CCR5, CSF1R, CD86, CD8A, and LCP2. *In vitro* qRT-PCR experiments were additionally performed to provide orthogonal experimental validation of SLC7A11, GPX4, ACSL4, and HMOX1 in 786-O versus RPTEC cells and of CCR5, CSF1R, and CD86 in a macrophage conditioned-medium model. The study therefore evaluates diagnostic and immune-microenvironment associations without inferring that these five genes directly regulate iron metabolism, lipid peroxidation, or the GPX4/SLC7A11 axis.

## 2 Materials and methods

### 2.1 Data sources and verification

The study used three public datasets. GSE53757 (GPL570, Affymetrix Human Genome U133 Plus 2.0 Array) contains 144 samples, comprising 72 ccRCC tissues and 72 matched normal kidney tissues. GSE159115 was used for single-cell analysis; GSM4819725 (SI_18854, patient SS_2005) represents a male clear-cell RCC tumor profiled using 10x Genomics 3′ v2 chemistry. The official GSE159115 annotation file matched 1,665 H5 barcodes. TCGA-KIRC consisted of a TPM expression matrix containing 19,938 genes across 614 samples, including 542 primary tumors and 72 normal kidney tissues, together with clinical annotations.

### 2.2 Transcriptomic preprocessing and differential expression

The GSE53757 series matrix and GPL570 annotation were parsed and probes were mapped to gene symbols. When multiple probes mapped to one gene, the probe with the highest mean expression was retained, yielding 22,189 genes across 144 samples. Linear signals were transformed using log2 (x + 1). ccRCC and normal tissues were compared using two-sided Welch t-tests followed by Benjamini-Hochberg (BH) correction. Significant differentially expressed genes (DEGs) were defined as adjusted p < 0.05 and |log2 fold change| > 0.5; preprocessing and sample-level quality-control diagnostics.

### 2.3 Hierarchical co-expression clustering

The 5,200 genes with the highest median absolute deviation were analyzed using 1 − |Pearson r| as the distance metric and Ward hierarchical clustering, and were partitioned into 20 modules. The first singular-value-decomposition component was used as the module eigengene. Module-phenotype associations were assessed by Spearman correlation, and module membership (MM) and gene significance (GS) were calculated for gene prioritization. Because this workflow did not use the standard WGCNA soft-threshold adjacency matrix, topological overlap matrix, or dynamic tree cutting, and because the maximum scale-free fit was R^2^ = 0.13, the analysis is described as hierarchical co-expression clustering rather than standard WGCNA (Langfelder and Horvath, 2008).

### 2.4 Functional enrichment and protein-protein interaction analysis

Significant DEGs within the hub module were ranked by MM × |GS|, and the top 250 genes were analyzed with Enrichr through gseapy for Gene Ontology Biological Process, KEGG, and WikiPathways enrichment (Kuleshov et al., 2016). A STRING v12 protein-protein interaction (PPI) network was then constructed using a minimum interaction score of 0.4, and hub position was summarized by degree centrality (Szklarczyk et al., 2023). Tumor-only Spearman correlations between the five hub genes and 12 canonical ferroptosis genes were compared across GSE53757 and TCGA-KIRC with BH correction.

### 2.5 MCP-counter immune scoring

Immune-cell scores were estimated with MCP-counter using the published transcriptomic marker framework (Becht et al., 2016). Scores were treated as expression-based estimates rather than direct cell-fraction measurements and were cross-checked against single-sample gene-set enrichment analysis (ssGSEA). To reduce circularity, each hub gene was removed from the relevant immune signature before tumor-only Spearman correlation analysis, and p values were adjusted using the BH method. An ancillary leave-one-out CD8 marker score was retained for the cross-cohort summary in Figure 1D.

**FIGURE 1:**
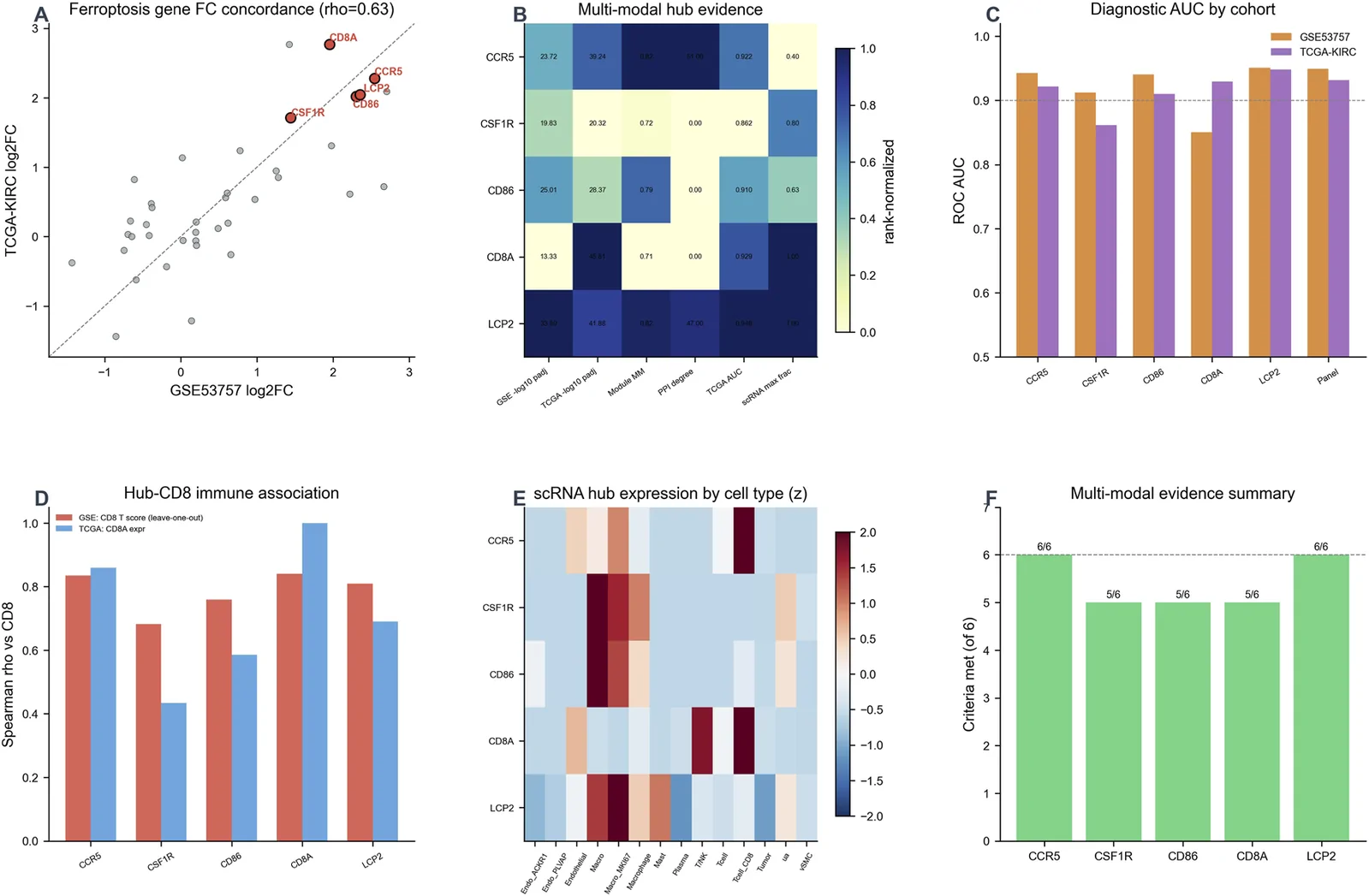
Cross-dataset integration. **(A)** Concordance of ferroptosis-gene log2FC values between GSE53757 and TCGA-KIRC. **(B)** Multi-modal evidence heatmap for the five hub genes across transcriptomic, module, PPI, single-cell, and TCGA analyses. **(C)** Diagnostic AUC comparison between GSE53757 and TCGA-KIRC. **(D)** Cross-cohort comparison of hub-gene associations with CD8-related immunity. **(E)** Single-cell hub-gene expression z-score heatmap. **(F)** Summary of six evidence criteria.

### 2.6 Single-cell analysis

The 10× H5 matrix for GSM4819725 was analyzed with scanpy. Cells with fewer than 200 detected genes or mitochondrial transcript proportions ≥25% were excluded. Counts were normalized to a target sum of 10,000 and log1p transformed. Two thousand highly variable genes were selected, followed by 50-component PCA, k-nearest-neighbor graph construction (n_neighbors = 15, n_pcs = 30), Leiden clustering (resolution = 0.6), and UMAP visualization. Official GSE159115 annotations were merged by barcode; cells lacking official annotation were assigned using classical marker-gene scores. Diffusion mapping and diffusion pseudotime were used only as exploratory ordering across the full mixed-cell population and were not interpreted as a lineage-differentiation trajectory. Ligand-receptor interaction scores were calculated from cell-type mean expression.

### 2.7 TCGA-KIRC validation

TCGA-KIRC TPM values were transformed using log2 (x + 1). Tumor and normal tissues were compared for the five hub genes. Diagnostic performance was assessed using ROC-AUC for each gene and for a five-gene panel defined as the mean of standardized gene-expression z scores. Overall survival was explored using Kaplan-Meier curves, log-rank tests, and Cox proportional-hazards models with a penalizer of 0.1. Associations with stage and grade were assessed using chi-square tests, and Spearman correlations were calculated with immune markers including CD8A, PDCD1, and CD68.

### 2.8 qRT-PCR experimental validation

For the two-model qRT-PCR experiments, relative expression was quantified using the comparative 2^−ΔΔCt^ approach, with GAPDH as the reference gene and RPTEC cells or M0 macrophages as the respective calibrators (Livak and Schmittgen, 2001). Three independent biological replicates per group were measured for SLC7A11, GPX4, ACSL4, and HMOX1 in 786-O and RPTEC cells and for CCR5, CSF1R, and CD86 in THP-1-derived macrophages maintained as M0 controls or exposed to 786-O-conditioned medium (786-O-CM). Inferential comparisons were performed on ΔCt values rather than on exponentiated fold-change values. Figure 2 presents the experimentally acquired *in vitro* qRT-PCR measurements and their statistical analysis.

**FIGURE 2:**
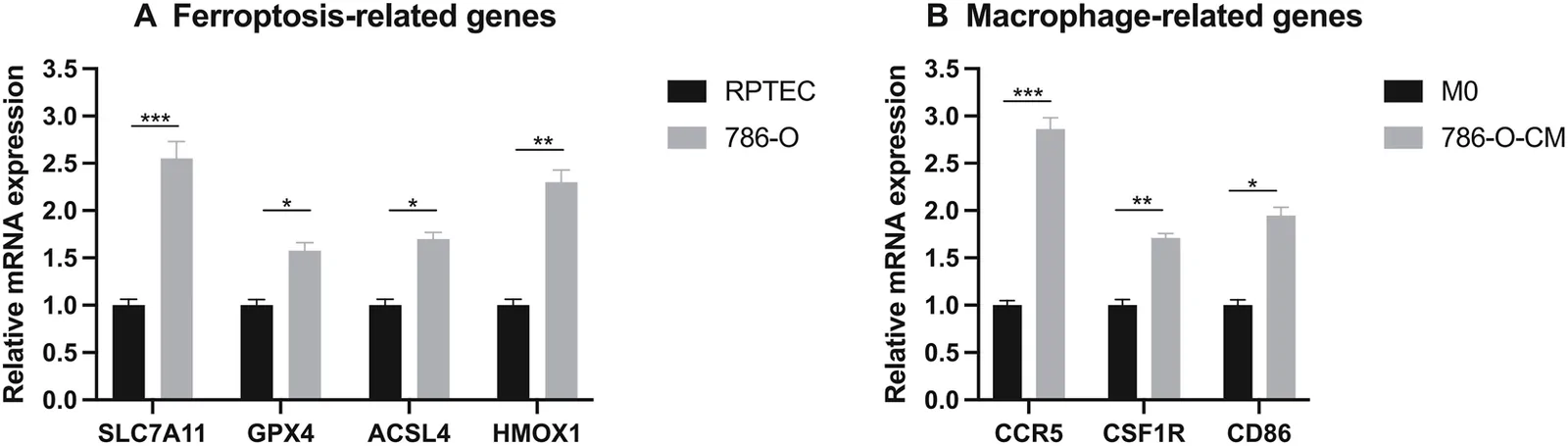
*In vitro* qRT-PCR validation in tumor-cell and macrophage models. **(A)** Relative mRNA expression of SLC7A11, GPX4, ACSL4, and HMOX1 in RPTEC and 786-O cells. **(B)** Relative mRNA expression of CCR5, CSF1R, and CD86 in THP-1-derived M0 macrophages and macrophages exposed to 786-O-conditioned medium (786-O-CM). Relative expression was calculated by the 2^–ΔΔCt^ method using GAPDH as the reference gene and the corresponding control group as the calibrator. Bars show mean ± SD for three independent biological replicates per group. Two-sided Welch *t*-tests were performed on ΔCt values. *p < 0.05, **p < 0.01, and ***p < 0.001.

### 2.9 Statistical analysis

Analyses were performed in Python 3.12 using numpy, pandas, scipy, scikit-learn, lifelines, scanpy, and gseapy. Continuous variables were compared using two-sided Welch t-tests or Mann-Whitney U tests as appropriate; multi-group comparisons used Kruskal-Wallis tests. Categorical variables were analyzed using chi-square or Fisher exact tests. Multiple testing was controlled using the BH procedure. Two-sided p < 0.05 was considered statistically significant.

## 3 Results

### 3.1 Differential expression and ferroptosis-gene enrichment in GSE53757

Among 22,189 genes, 8,650 DEGs were identified, including 3,722 upregulated and 4,928 downregulated genes (adjusted p < 0.05, |log2FC| > 0.5). Of 35 FerrDb v2 ferroptosis genes, 34 were detected in GSE53757 and 13 were significantly upregulated DEGs: SLC7A11, HMOX1, ALOX5, TP53, CDKN1A, MDM2, BAX, BCL2, PMAIP1, FANCD2, BRCA1, BRCA2, and EGFR. Hypergeometric enrichment was significant (p = 2.3 × 10^−3^), supporting ferroptosis-related transcriptional remodeling in ccRCC. Cross-cohort hub-gene/canonical-ferroptosis-gene correlation patterns were concordant (rho = 0.65, p = 2.0 × 10^−8^). Normalization aligned sample distributions and preserved tumor-normal separation on PCA. The five target hub genes were also significantly upregulated: CCR5 (log2FC = 2.54, adjusted p = 1.9 × 10^−24^), LCP2 (2.35, 2.6 × 10^−34^), CD86 (2.30, 9.9 × 10^−26^), CD8A (1.95, 4.6 × 10^−14^), and CSF1R (1.44, 1.5 × 10^−20^) (Figures 3A–F).

**FIGURE 3:**
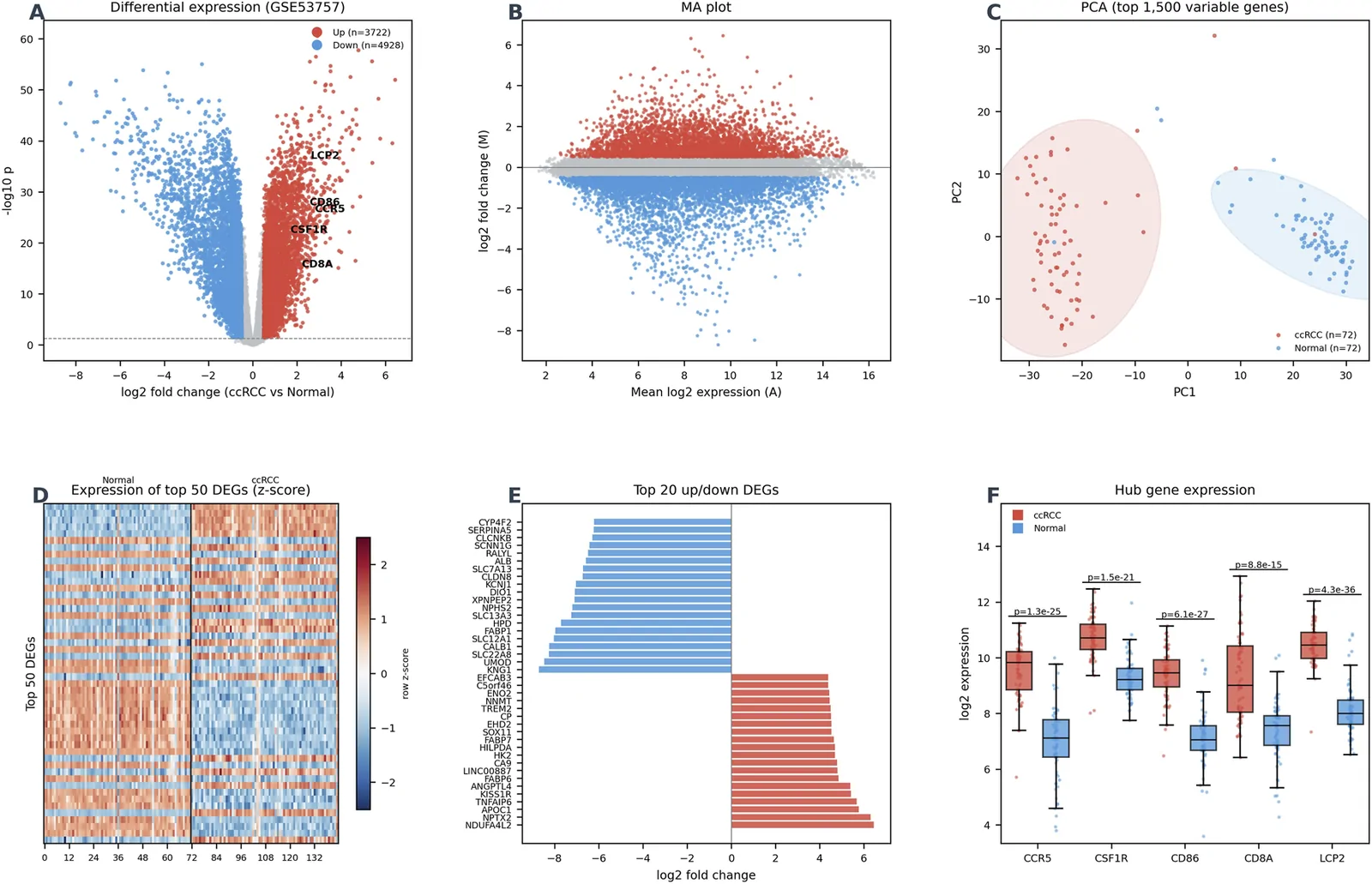
Differential expression analysis in GSE53757 (72 ccRCC versus 72 normal kidney tissues; GPL570). **(A)** Volcano plot, with significantly upregulated and downregulated genes defined by adjusted p < 0.05 and |log2FC| > 0.5; the five hub genes are annotated. **(B)** MA plot. **(C)** PCA using the top 1,500 highly variable genes; ellipses indicate 95% confidence regions. **(D)** Row-z-score heatmap of the top 50 DEGs. **(E)** Bidirectional bar plot of the top 20 upregulated and downregulated genes. **(F)** Expression boxplots for CCR5, CSF1R, CD86, CD8A, and LCP2 using two-sided Welch t-tests.

### 3.2 Co-expression modules and functional enrichment

Ward hierarchical clustering identified 20 co-expression modules. The maximum scale-free topology fit was R^2^ = 0.13; therefore, the analysis was not designated as standard WGCNA. Module M03 showed a strong positive association with the ccRCC phenotype (r = 0.80, p = 5.1 × 10^−33^), and all five hub genes were located in M03 (MM = 0.71–0.82; GS = 0.61–0.78). Significant M03 DEGs were enriched in immune-related biological processes, including regulation of interleukin-1β production (adjusted p = 2.4 × 10^−10^), defense response to symbiont (4.0 × 10^−10^), and T-cell activation (2.5 × 10^−7^). KEGG enrichment included chemokine signaling (3.2 × 10^−8^), phagosome (4.0 × 10^−7^), and natural-killer-cell-mediated cytotoxicity (4.0 × 10^−7^) (Figures 4A–F).

**FIGURE 4:**
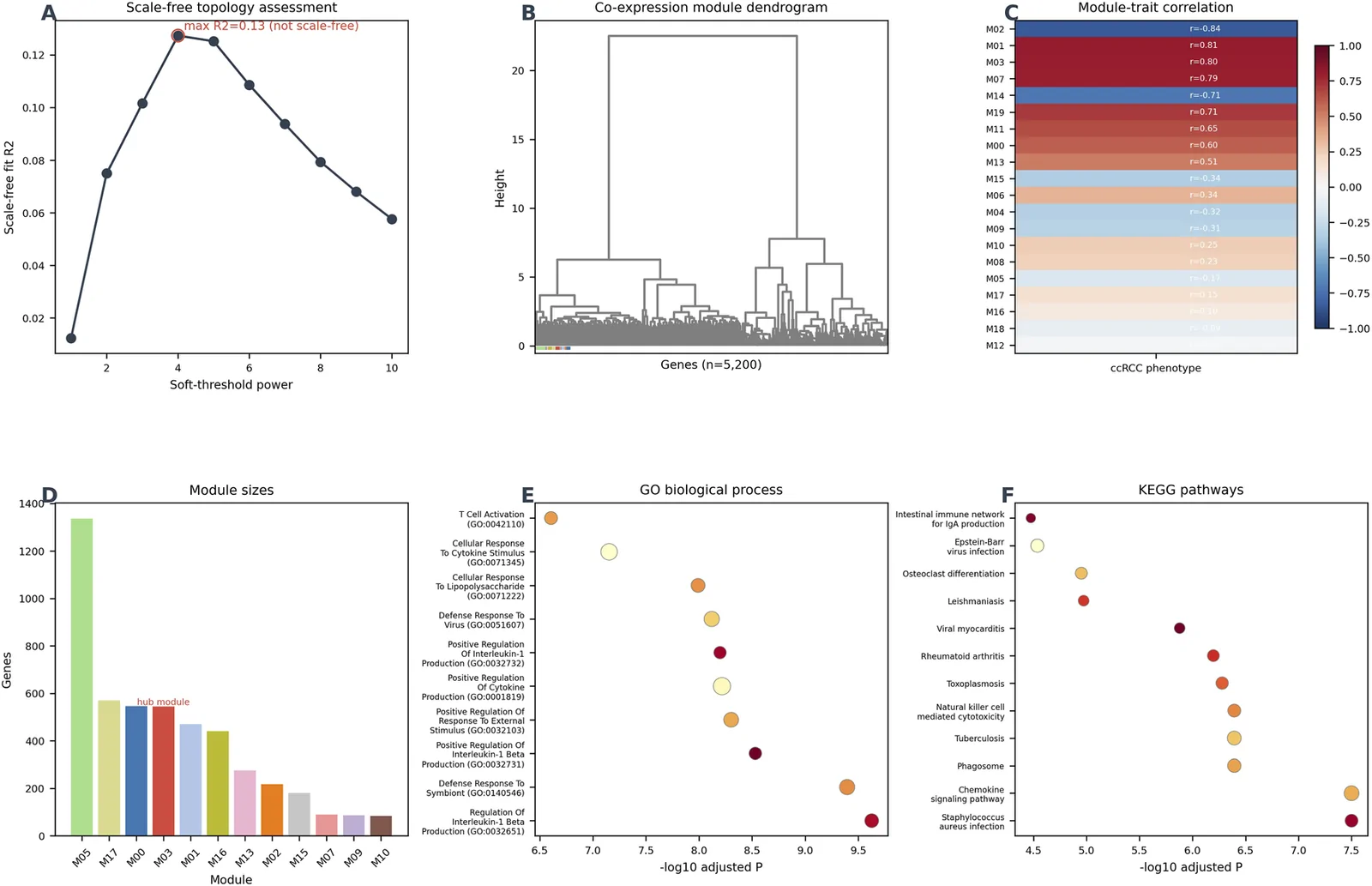
Co-expression modules and enrichment in GSE53757 using 1 − |r| Ward hierarchical clustering rather than standard WGCNA. **(A)** Scale-free topology assessment; the maximum soft-threshold fit was R^2^ = 0.13. **(B)** Ward dendrogram of 5,200 highly variable genes, with module assignment shown below. **(C)** Module-phenotype correlation heatmap; M03 showed r = 0.80. **(D)** Module sizes. **(E)** GO Biological Process enrichment bubble plot. **(F)** KEGG pathway enrichment bubble plot.

### 3.3 MCP-counter immune scores

Paired MCP-counter scores for cytotoxic lymphocytes, monocytic lineage, endothelial cells, NK cells, T cells, myeloid dendritic cells, and fibroblasts were higher in ccRCC (all FDR <0.001), whereas B-lineage and neutrophil scores were lower (FDR <0.05). After leave-one-out scoring in tumor samples, CD8A correlated with the cytotoxic-lymphocyte score (rho = 0.66, FDR = 1.2 × 10^−9^), and CSF1R correlated with the monocytic-lineage score (rho = 0.48, FDR = 6.7 × 10^−5^). MCP-counter and ssGSEA estimates were highly concordant across all nine populations (rho = 0.88–0.99; Figures 5A–F).

**FIGURE 5:**
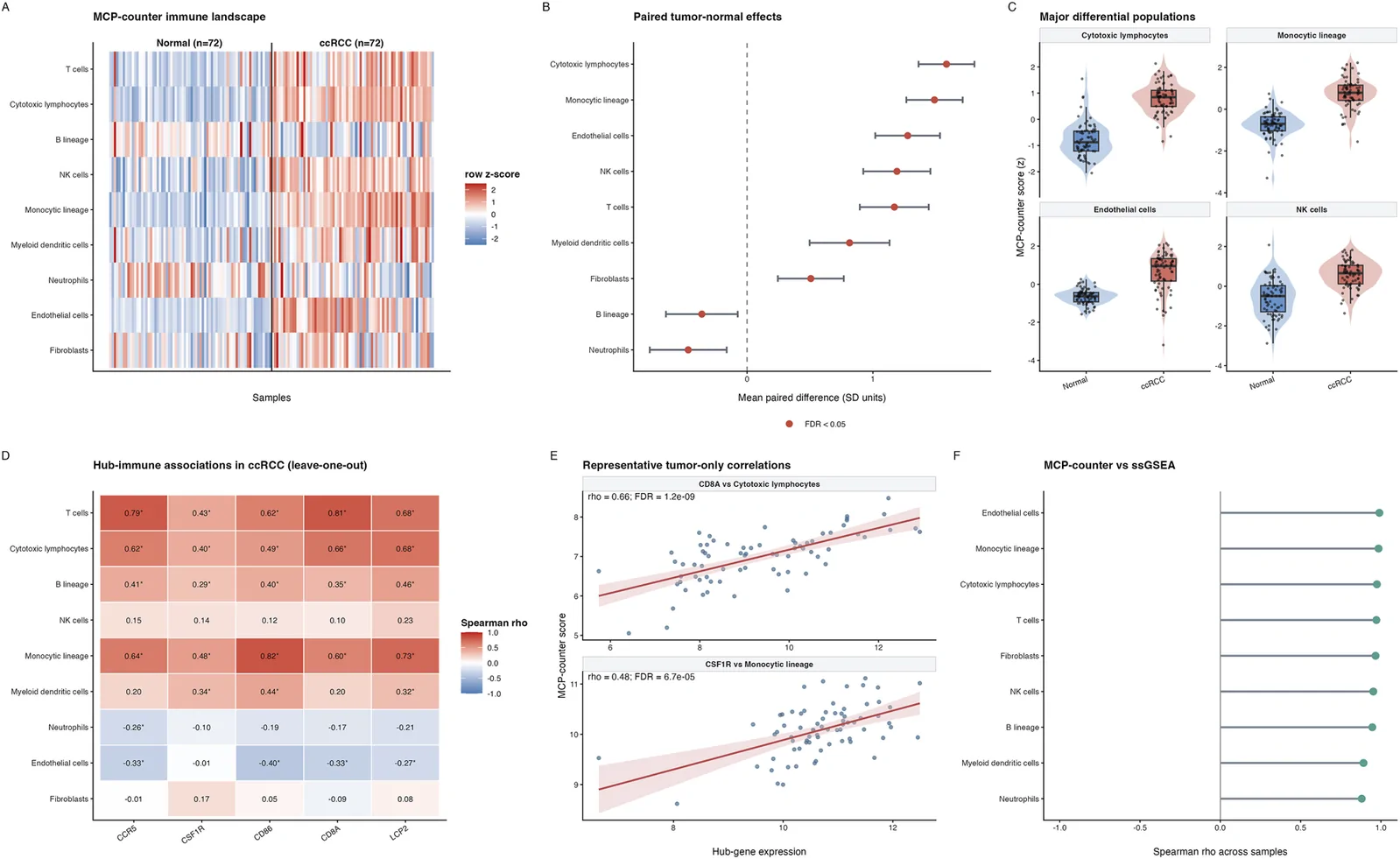
MCP-counter immune scoring and cross-method concordance in GSE53757. Scores are expression-based estimates rather than direct cell-fraction measurements. **(A)** Sample-wise MCP-counter landscape for nine immune and stromal populations. **(B)** Paired tumor-normal effects with 95% confidence intervals. **(C)** Violin and box plots for the four largest paired effects. **(D)** Tumor-only Spearman correlation heatmap between hub genes and leave-one-out immune scores. **(E)** Representative CD8A-cytotoxic-lymphocyte and CSF1R-monocytic-lineage correlations. **(F)** MCP-counter/ssGSEA concordance across the nine populations.

### 3.4 PPI network and hub-gene position

The STRING network contained 1,431 edges. High-degree immune nodes included PTPRC, TYROBP, ITGB2, CCR5, LCP2, IL10RA, and TLR2. CCR5 (degree = 51) and LCP2 (degree = 47) ranked among the top ten nodes, supporting their central position within the immune-associated interaction network (Figures 6A–F).

**FIGURE 6:**
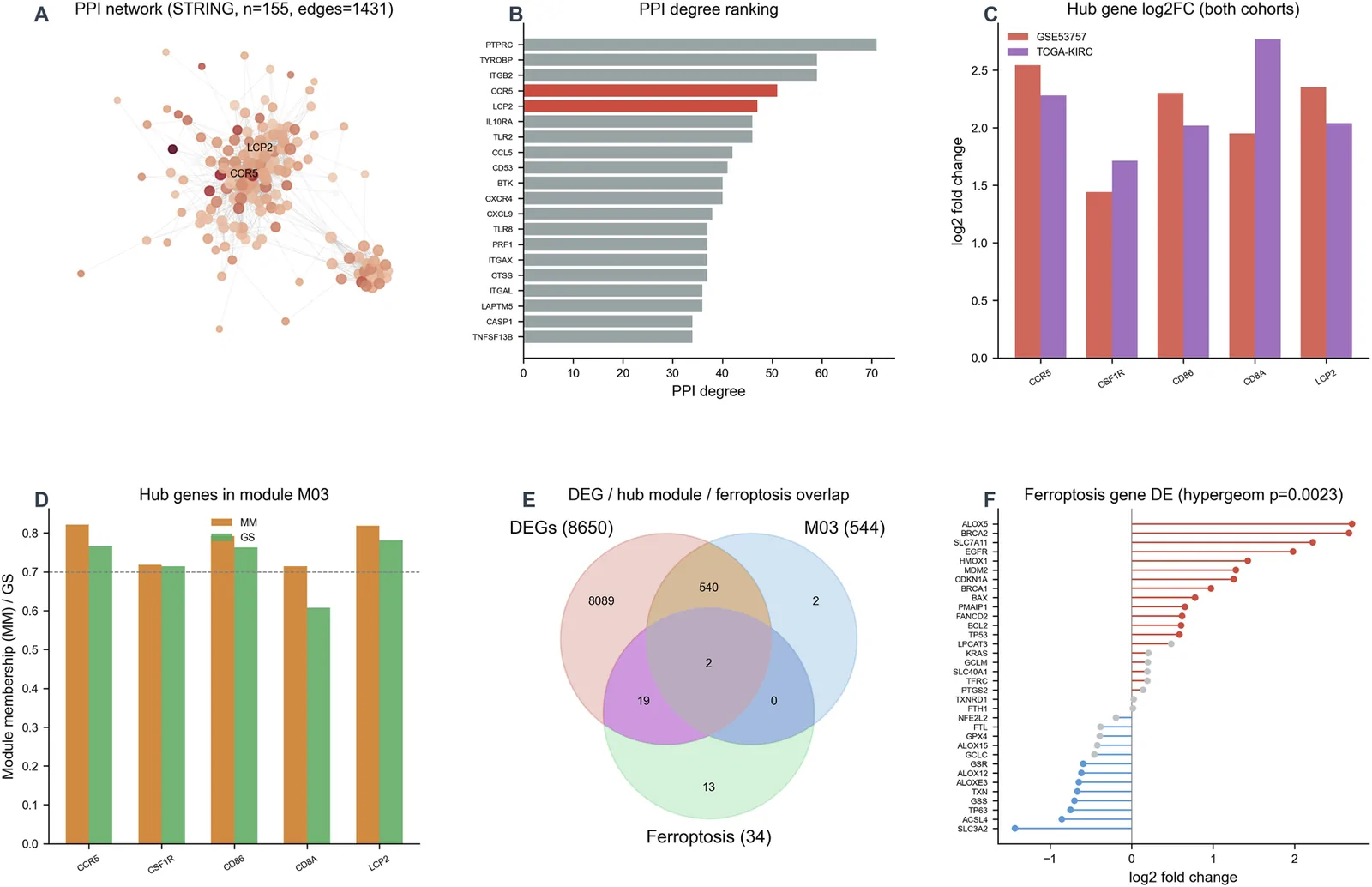
PPI network and hub-gene characteristics. **(A)** STRING PPI network containing 1,431 edges; node color represents log2FC. **(B)** Top 20 genes by degree centrality, with CCR5 and LCP2 highlighted. **(C)** Comparison of hub-gene log2FC values between GSE53757 and TCGA-KIRC. **(D)** Module membership and gene significance of the hub genes. **(E)** Venn diagram of DEGs, M03-module genes, and ferroptosis genes. **(F)** Lollipop plot showing differential expression of 34 detected ferroptosis genes.

### 3.5 Single-cell localization

Quality control removed 21 of 1,704 cells and retained 1,683 cells from GSM4819725 (Figures 7A,F). Official annotations were available for 1,665 cells, including 992 tumor cells, 517 macrophages, 59 CD8 T cells, 23 vascular smooth-muscle cells, 23 unclassified cells, 20 endothelial cells, 8 plasma cells, and 5 mast cells. After marker-based assignment of the 18 unannotated cells, the major composition was 995 tumor cells (59.1%), 517 macrophages (30.7%), and 59 CD8 T cells (3.5%) (Figures 7A–F). CCR5, CSF1R, and CD86 were enriched in macrophage-lineage populations; their positive-cell proportions across Macro, Macro_MKI67, and Macrophage populations ranged from 11.0% to 40.0%, 66.7%–80.0%, and 33.3%–62.7%, respectively. CD8A showed the highest positivity in T/NK cells (100%, n = 3) and CD8 T cells (89.8%, n = 59). LCP2 was also prominent in macrophage-lineage populations (Macro_MKI67 100%, Macrophage 77.8%, Macro 71.6%). Exploratory diffusion pseudotime showed dynamic patterns for LCP2 and CD8A, but because the ordering crossed tumor, macrophage, T/NK, and endothelial compartments, it was not interpreted as a single-lineage differentiation trajectory (Figures 8A–F).

**FIGURE 7:**
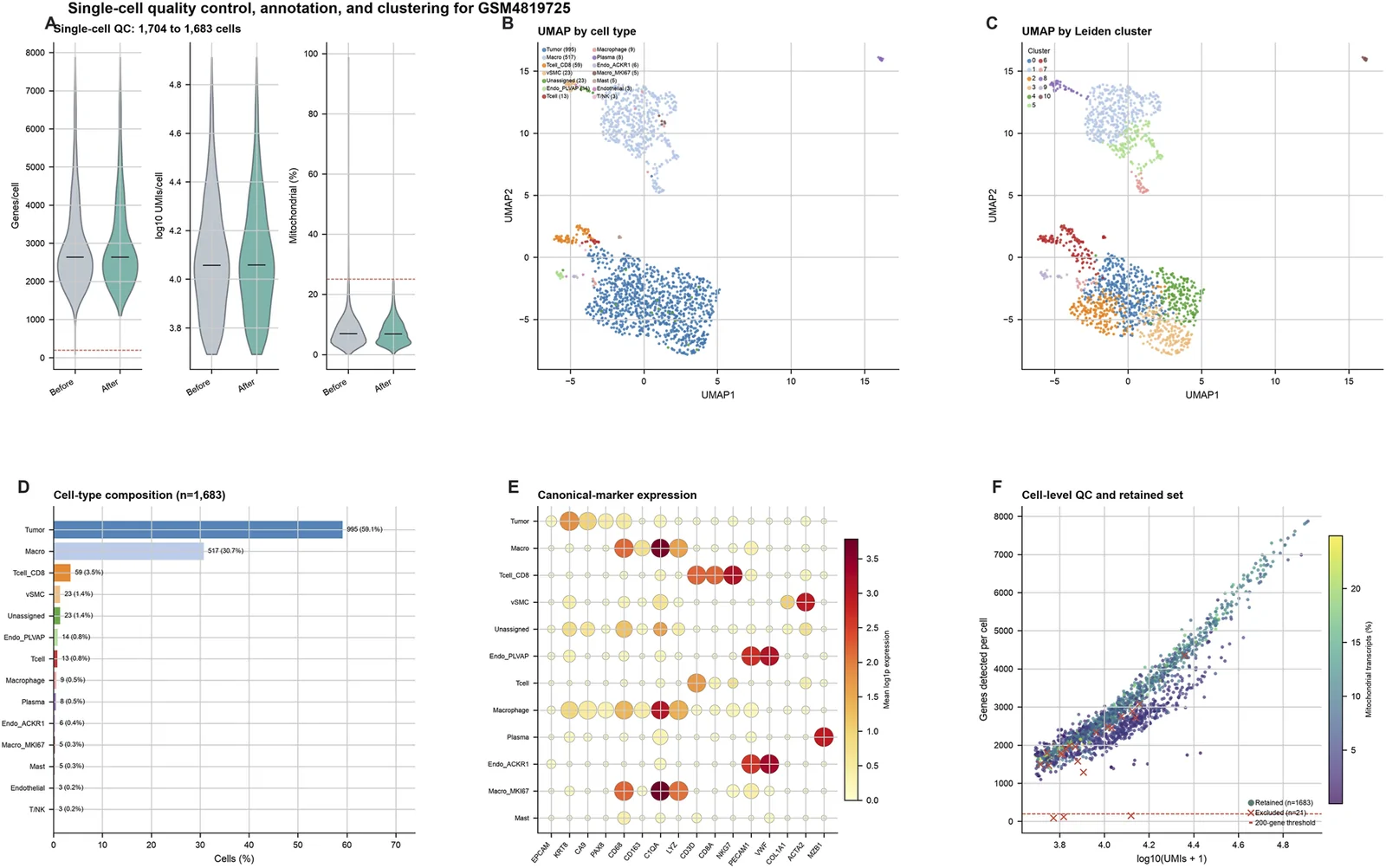
Single-cell quality control, annotation, and clustering for GSM4819725 (ccRCC; SI_18854). **(A)** Violin plots of genes per cell, log10 UMI counts, and mitochondrial-transcript percentages before and after filtering. **(B)** UMAP colored by cell type. **(C)** UMAP colored by Leiden cluster. **(D)** Cell-type composition (n = 1,683). **(E)** Dot plot of canonical marker genes; dot size indicates the proportion of expressing cells and color indicates mean expression. **(F)** Cell-level QC scatter plot showing retained and excluded cells relative to the filtering thresholds.

**FIGURE 8:**
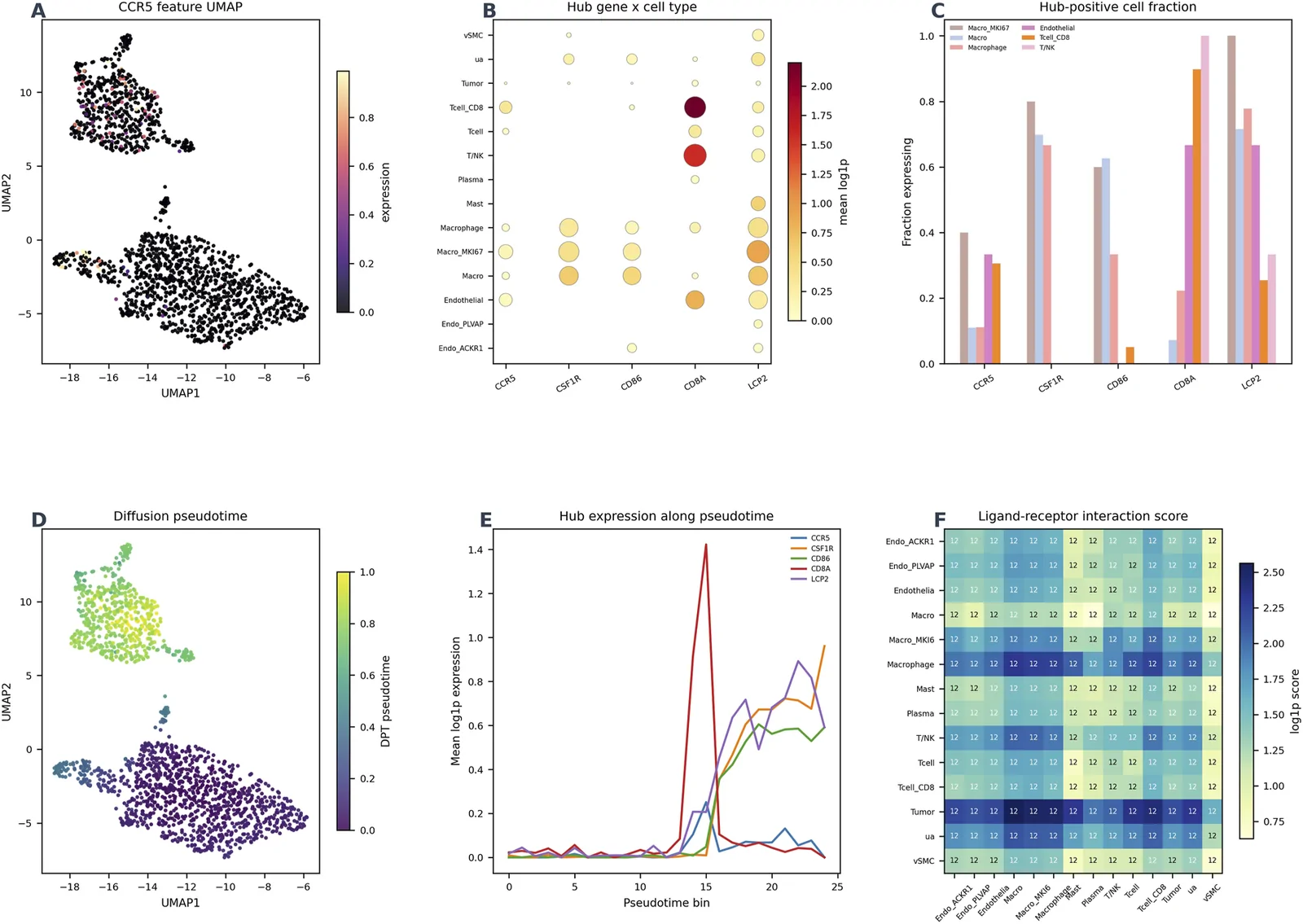
Single-cell localization of hub genes and exploratory pseudotime ordering. **(A)** CCR5 feature UMAP. **(B)** Dot plot of hub-gene expression across cell types. **(C)** Hub-gene positive-cell proportions by cell type. **(D)** Diffusion-pseudotime UMAP; the ordering spans tumor, macrophage, T/NK, endothelial, and other populations and is not interpreted as a differentiation trajectory. **(E)** Hub-gene expression trends along the exploratory ordering. **(F)** Ligand-receptor interaction score heatmap.

### 3.6 TCGA-KIRC validation

TCGA-KIRC independently confirmed significant upregulation of all five hub genes in 542 tumors versus 72 normal kidney tissues (log2FC range 1.71–2.77; all adjusted p < 1.5 × 10^−20^). Diagnostic ROC analysis yielded AUCs of 0.948 for LCP2, 0.932 for the five-gene panel, 0.929 for CD8A, 0.922 for CCR5, 0.910 for CD86, and 0.862 for CSF1R. In GSE53757, single-gene AUCs ranged from 0.86 to 0.95. Hub-gene expression was positively correlated with immune markers including CD8A, GZMB, PDCD1, and CD68. In the survival dataset (n = 538; 173 events), high-versus-low expression groups for the five genes showed no significant overall-survival differences (log-rank p = 0.41–0.93). In multivariable Cox analysis, age (HR = 1.02 per year, p = 3.5 × 10^−4^) and advanced stage (HR = 2.76, p = 9.0 × 10^−14^) were significant, whereas the five-gene panel had no independent prognostic value (Figures 1A–F, 9A–F).

**FIGURE 9:**
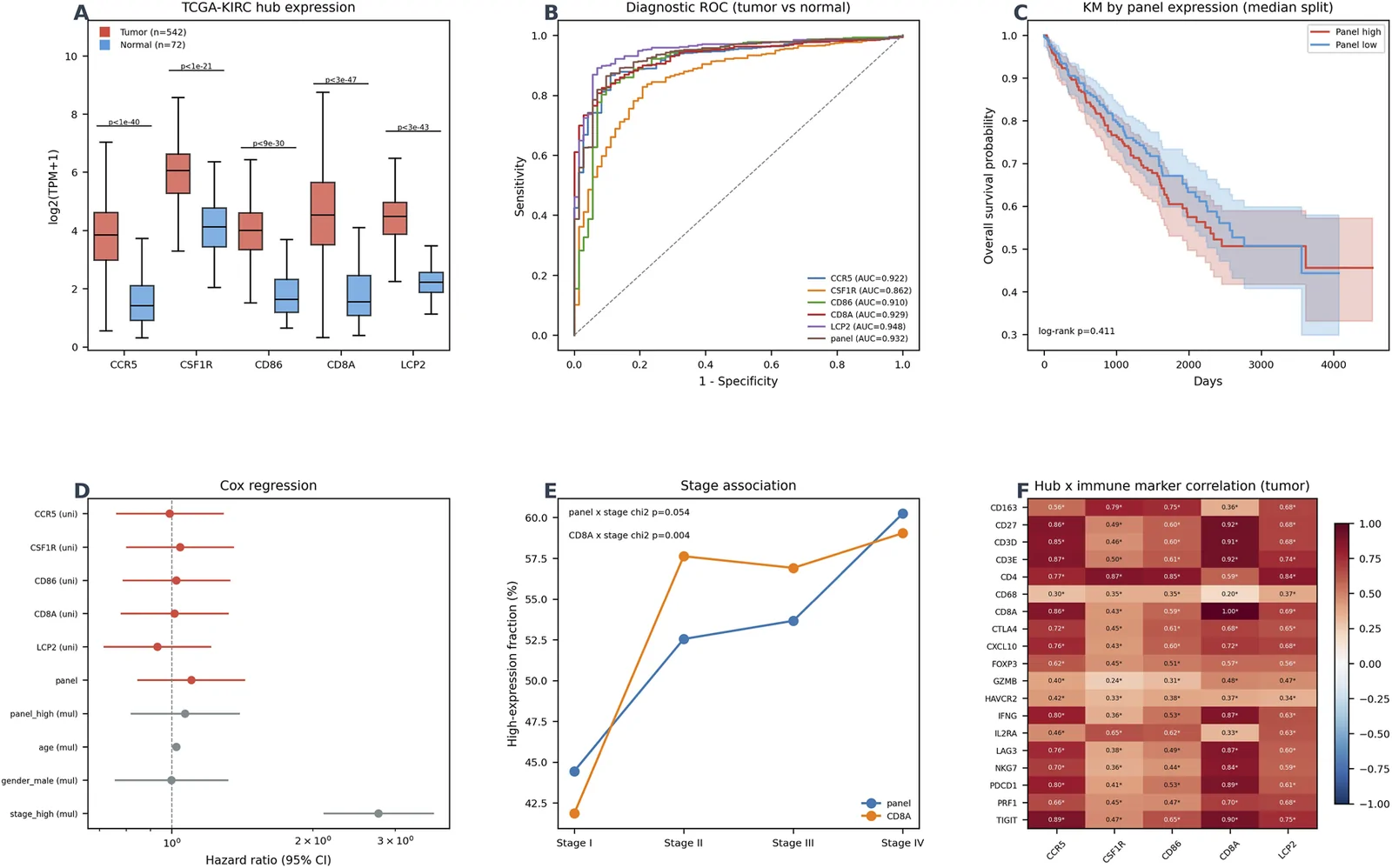
Independent TCGA-KIRC validation. **(A)** Tumor-versus-normal expression boxplots for the five hub genes (542 versus 72). **(B)** Diagnostic ROC curves, including LCP2 AUC = 0.948 and five-gene panel AUC = 0.932. **(C)** Kaplan-Meier curve for the panel (log-rank p = 0.41). **(D)** Cox-regression forest plot. **(E)** Proportions of high panel and high CD8A expression across stages. **(F)** Correlation heatmap between hub genes and immune markers.

### 3.7 qRT-PCR experimental validation results

The *in vitro* qRT-PCR experiment included three independent biological replicates per group for seven genes across the two experimental models. In RPTEC versus 786-O cells, relative expression (2^−ΔΔCt^; mean ± SD) was 1.00 ± 0.06 versus 2.55 ± 0.18 for SLC7A11, 1.00 ± 0.06 versus 1.58 ± 0.08 for GPX4, 1.00 ± 0.06 versus 1.70 ± 0.07 for ACSL4, and 1.00 ± 0.06 versus 2.30 ± 0.13 for HMOX1. In M0 versus 786-O-CM macrophages, the corresponding values were 1.00 ± 0.05 versus 2.86 ± 0.12 for CCR5, 1.00 ± 0.06 versus 1.71 ± 0.05 for CSF1R, and 1.00 ± 0.06 versus 1.95 ± 0.09 for CD86. Welch t-tests on ΔCt values indicated significant differences for all seven genes (Figure 2). These experimentally acquired measurements show higher expression of the four ferroptosis-related genes in 786-O cells and of the three macrophage markers after 786-O-CM exposure.

## 4 Discussion

This study integrates bulk transcriptomics, hierarchical co-expression analysis, MCP-counter immune scoring, PPI analysis, single-cell RNA sequencing, and independent TCGA-KIRC validation to characterize CCR5, CSF1R, CD86, CD8A, and LCP2 as immune-microenvironment-associated hub genes in ccRCC. These genes are not canonical ferroptosis effectors. Their relevance to ferroptosis arises from their coexistence with a ferroptosis-enriched transcriptional state and from network-level association within the ccRCC molecular landscape.

The pathway-level ferroptosis signal in GSE53757 was statistically supported by enrichment of FerrDb genes among the differentially expressed genes, including SLC7A11, HMOX1, ALOX5, TP53, CDKN1A, MDM2, BAX, BCL2, PMAIP1, FANCD2, BRCA1, BRCA2, and EGFR. Independent work has likewise identified ferroptosis-associated molecular heterogeneity in ccRCC and has shown that ferroptosis-related gene patterns can stratify tumors with distinct biological and immune characteristics (Wang et al., 2024). A recent review further summarizes the mechanistic relevance of iron handling, lipid peroxidation, cystine transport, and antioxidant defense in ccRCC, supporting the biological plausibility of the pathway-level signal observed here (Pan et al., 2024).

More recent mechanistic work also supports crosstalk between ferroptosis-related metabolism and the immune microenvironment. LPCAT3, a phospholipid-remodeling enzyme with direct relevance to ferroptotic lipid substrates, has been linked to immune infiltration and ccRCC biology (Feng et al., 2025). These observations reinforce a two-layer interpretation of our findings: canonical ferroptosis genes define the metabolic cell-death axis, whereas CCR5, CSF1R, CD86, CD8A, and LCP2 primarily report the immune compartment that accompanies that axis. The present results therefore support a ferroptosis-associated immune context rather than direct ferroptosis regulation by the five hub genes. The cross-cohort correlation concordance supports a reproducible transcriptomic association but not a direct mechanism.

The co-expression analysis is also important methodologically. The data structure did not satisfy the scale-free fit expected for standard WGCNA; accordingly, the procedure was defined as Ward hierarchical co-expression clustering. M03 showed a strong positive phenotype association and contained all five hub genes. Its enrichment for T-cell activation, chemokine signaling, phagosome biology, and NK-cell cytotoxicity demonstrates that the dominant functional identity of the module is immune rather than ferroptotic. Related ccRCC studies using multi-omics, machine learning, and single-cell analysis have similarly shown that regulated-cell-death signatures frequently intersect with immune and stromal programs (Liu et al., 2024).

The single-cell analysis provides a cell-type-resolved explanation for the bulk-tissue hub signals. CCR5, CSF1R, CD86, and LCP2 were concentrated mainly in macrophage-lineage populations, while CD8A localized primarily to CD8 T/T-NK compartments. This pattern is biologically coherent with current single-cell investigations demonstrating that death-program-associated transcriptional states in ccRCC are embedded within complex intercellular networks involving myeloid, lymphoid, epithelial, and stromal populations (Xu et al., 2024). Consequently, increased bulk expression of these genes should not be interpreted as evidence that malignant epithelial cells themselves overexpress every hub gene to the same degree.

The MCP-counter correlations further support this interpretation. After leave-one-out scoring, CD8A remained correlated with the cytotoxic-lymphocyte score and CSF1R with the monocytic-lineage score, while MCP-counter and ssGSEA estimates were concordant, reducing method-specific and self-correlation bias. Prior ccRCC co-expression work has independently identified CCR5 and CD8A among genes associated with CD8-positive T-cell infiltration (Lin et al., 2020). In parallel, transcriptomic and RT-qPCR studies of ccRCC have reported increased CD86 expression among immune-associated differentially expressed genes, supporting its interpretation as part of an antigen-presenting/myeloid immune program rather than a ferroptosis enzyme (Li et al., 2021).

CSF1R deserves similar caution. It is a lineage-defining receptor in monocyte/macrophage biology, and recent ccRCC work has directly implicated CSF1-CSF1R signaling in tumor-macrophage crosstalk (Li et al., 2026). Therefore, the high CSF1R signal observed in bulk tumor tissue is plausibly explained by tumor-associated myeloid content. This is consistent with the single-cell localization in the present analysis and illustrates why immune-cell abundance must be separated conceptually from tumor-cell-intrinsic expression when interpreting bulk transcriptomes.

Independent TCGA-KIRC validation strengthened the diagnostic component of the study. All five hub genes were significantly upregulated in tumor relative to normal tissue, and the ROC analysis yielded high AUCs across individual genes and the continuous five-gene z-score panel. LCP2 showed the highest single-gene AUC, while the panel retained high discrimination. The validation strategy uses a continuous five-gene expression score, providing a reproducible analytic definition across transcriptomic cohorts.

In contrast, the survival findings were negative. Neither the individual hub genes nor the combined panel showed convincing overall-survival separation in TCGA-KIRC, and the panel did not retain an independent prognostic effect in multivariable analysis. This negative result is informative because it constrains the scope of the conclusions. The five-gene panel should therefore be described as a candidate diagnostic and immune-microenvironment marker set rather than a validated prognostic signature.

From a biological perspective, the findings suggest that the strongest utility of the five genes may lie in describing immune architecture rather than ferroptotic sensitivity *per se*. A tumor with high CCR5, CSF1R, CD86, CD8A, and LCP2 expression may reflect coordinated enrichment of myeloid and cytotoxic-lymphoid compartments. Whether such an immune-rich state is more or less susceptible to pharmacologic ferroptosis induction cannot be inferred from expression alone. Addressing that question would require perturbation experiments with ferroptosis inducers or inhibitors, lipid-ROS measurements, labile iron assays, GPX4/SLC7A11 manipulation, and rescue experiments with ferrostatin-1 or liproxstatin-1.

The qRT-PCR component provides experimental orthogonal validation of ferroptosis-related gene expression in 786-O versus RPTEC cells and of macrophage-lineage markers in the M0 versus 786-O-CM model. The analysis was based on experimentally acquired ΔCt values, from which ΔΔCt and 2^ΔΔCt^ relative-expression values were calculated; inferential testing was conducted on ΔCt values. The *in vitro* measurements showed significantly higher expression of SLC7A11, GPX4, ACSL4, and HMOX1 in 786-O cells and of CCR5, CSF1R, and CD86 in 786-O-CM-treated macrophages, extending the cell-type-resolved findings presented in Figure 8.

Several limitations should be acknowledged. First, the principal multi-omics evidence is transcriptomic and associative, so causal ferroptosis mechanisms cannot be inferred. Second, MCP-counter scores are expression-based estimates rather than direct cell-fraction measurements. Third, the single-cell analysis is based on a limited ccRCC sample and should be validated in larger independent single-cell cohorts. Fourth, the exploratory diffusion-pseudotime ordering spans multiple lineages and should not be interpreted as a differentiation trajectory. Fifth, batch, platform, and cohort differences may influence cross-dataset effect sizes even when directionality is concordant. Although the seven-gene qRT-PCR findings were obtained in two *in vitro* models, additional independent cell lines, primary samples, and mechanistic experiments would further strengthen their generalizability and biological interpretation.

## 5 Conclusion

Across GSE53757, GSE159115, and TCGA-KIRC, CCR5, CSF1R, CD86, CD8A, and LCP2 showed consistent upregulation, strong diagnostic discrimination, and distinct immune-cell localization in ccRCC. The evidence supports their classification as immune-microenvironment-associated hub biomarkers rather than direct ferroptosis drivers. Their relationship with ferroptosis is currently supported by DEG enrichment, co-expression context, and tumor-cell expression of SLC7A11, GPX4, ACSL4, and HMOX1. Future mechanistic studies should test lipid peroxidation, iron metabolism, and the GPX4/SLC7A11 axis directly.

## Data Availability

The original qRT-PCR data and other supporting data are available from the corresponding author upon reasonable request. The names of the repository/repositories and accession number(s) can be found in the article/Supplementary Material.
