# Supplementary material for: Immune microenvironment biomarkers linked to ferroptosis in clear cell renal cell carcinoma
Source: Front Genet. 2026 Oct 28;17:1938003. doi: 10.3389/fgene.2026.1938003 (PMC13752832; doi:10.3389/fgene.2026.1938003)
Supplement: Supplementary file 1 [file Supplementaryfile1.pdf]

Supplementary Figure S1

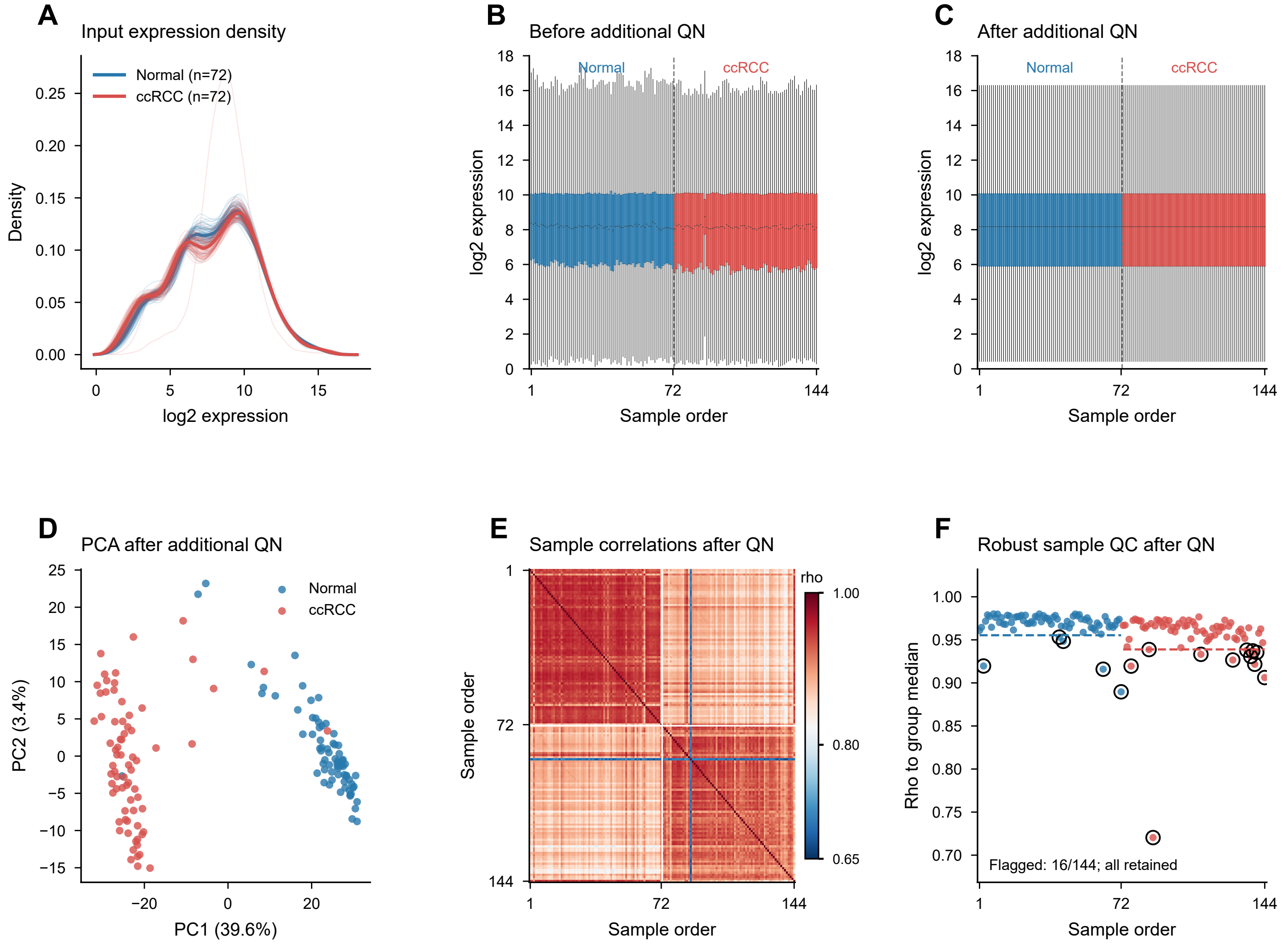

Supplementary Figure S1. Bulk-expression preprocessing and quality-control diagnostics for GSE53757. Diagnostics derive from 144 samples (72 ccRCC and 72 matched normal kidney tissues) and 22,189 mapped genes. The archived gene-level matrix was log2(x + 1)-transformed; an additional quantile normalization (QN) was performed only for the supplementary diagnostics in C-F. (A) Sample expression-density curves before additional QN; thick lines show group-median densities. (B,C) Sample box plots before and after additional QN. Boxes span the interquartile range, center lines are medians, and whiskers show minima and maxima. (D) PCA after additional QN using 1,500 genes selected by highest median absolute deviation in the archived input, with genes standardized before PCA. (E) Sample-sample Spearman correlations after additional QN; color limits are 0.65-1.00 and sample order is normal followed by ccRCC in B, C, E, and F. (F) Sample correlations to leave-one-out within-group median expression profiles after additional QN. Dashed lines mark group-specific median minus 3 times unscaled MAD thresholds; black rings identify flagged samples. Flags are diagnostic and no samples were removed. Blue denotes normal and red denotes ccRCC. Archived downstream differential-expression and other analysis inputs were unchanged.

Supplementary Figure S2

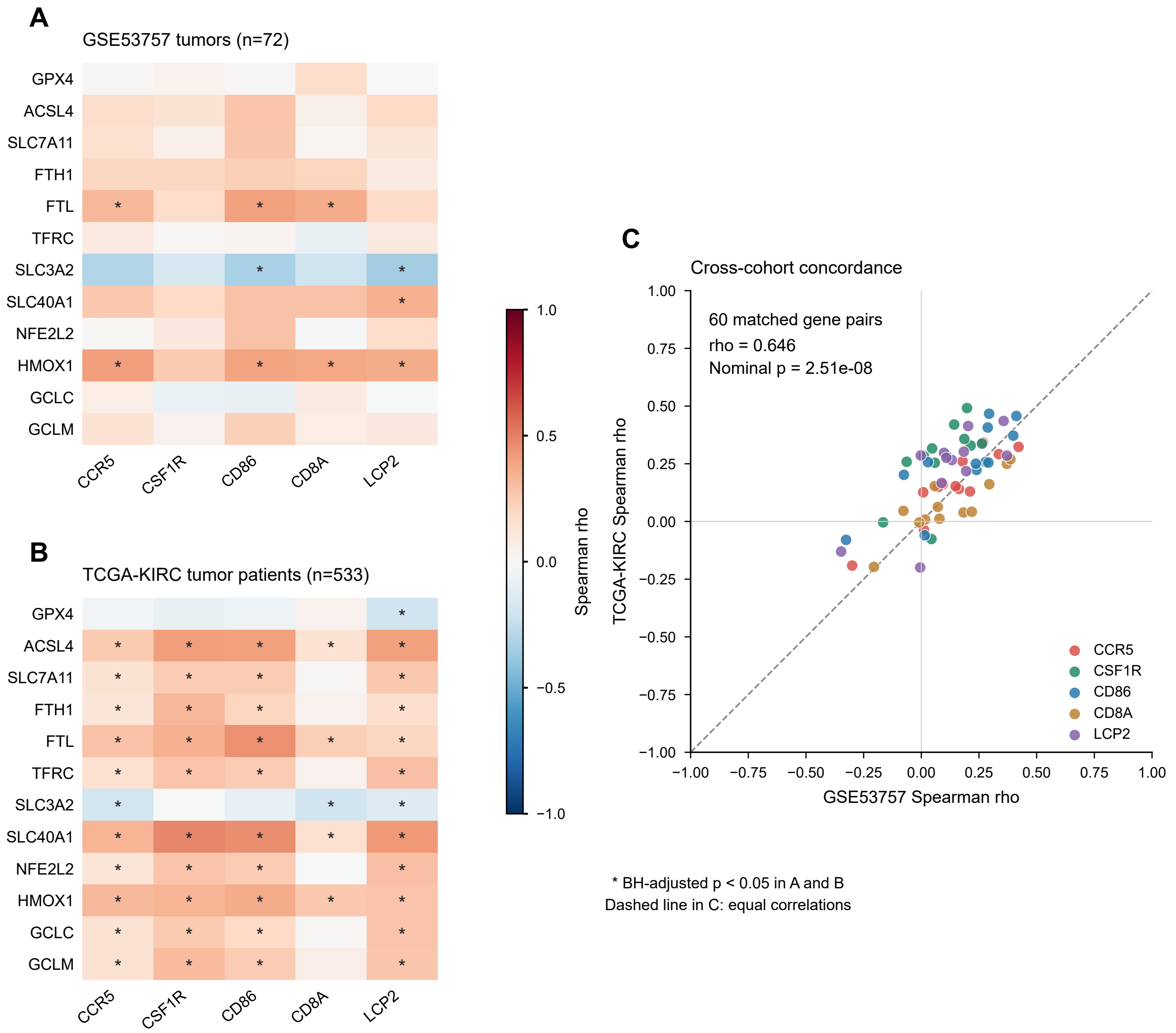

Supplementary Figure S2. Associations between immune-microenvironment hub genes and a 12-gene ferroptosis panel. The ferroptosis genes are GPX4, ACSL4, SLC7A11, FTH1, FTL, TFRC, SLC3A2, SLC40A1, NFE2L2, HMOX1, GCLC, GCLM. (A,B) Tumor-only Spearman correlations for CCR5, CSF1R, CD86, CD8A, and LCP2 in GSE53757 (72 tumor samples) and TCGA-KIRC (533 tumor patients), respectively. TCGA log2(TPM + 1) expression values from 542 tumor records (541 Primary Tumor and one Additional - New Primary record) were aggregated to one median expression profile per patient. Red and blue indicate positive and negative correlations, respectively. Asterisks denote BH-adjusted $p < 0.05$ across all 60 gene pairs within each cohort. (C) Concordance of the 60 matched correlation coefficients (Spearman rho = 0.646; nominal two-sided p = 2.51e-08). Points are colored by hub gene; the dashed line indicates equal correlations. The concordance p value is exploratory because gene pairs share genes and are not independent biological replicates.
